# A case report of familial 4q13.3 microdeletion in three individuals with syndromic intellectual disability

**DOI:** 10.1186/s12920-020-0711-4

**Published:** 2020-04-16

**Authors:** Živilė Maldžienė, Evelina M. Vaitėnienė, Beata Aleksiūnienė, Algirdas Utkus, Eglė Preikšaitienė

**Affiliations:** 1 Department of Human and Medical Genetics, Institute of Biomedical Sciences, Faculty of Medicine, Vilnius University, Santariškių st. 2, 08661 Vilnius, LT Lithuania; 2 Faculty of Medicine, Vilnius University, Vilnius, Lithuania

**Keywords:** 4q13.3 microdeletion, *ADAMTS3*, *ANKRD17*, *COX18*, Intellectual disability, Congenital anomalies

## Abstract

**Background:**

Interstitial 4q deletions are rare chromosomal alterations. Most of the previously reported deletions involving the 4q13.3 region are large chromosomal alterations with a common loss of band 4q21 resulting in marked growth restriction, severe intellectual disability, and absent or severely delayed speech. A microdeletion of 4q13.3 hasn’t been previously reported. We discuss the involvement of genes and the observed phenotype, comparing it with that of previously reported patients.

**Case presentation:**

We report on a 4q13.3 microdeletion detected in three affected individuals of a Lithuanian family. The clinical features of two affected children and their affected mother are very similar and include short stature, congenital heart defect, skeletal anomalies, minor facial anomalies, delayed puberty, and intellectual disability. Whole genome SNP microarray analysis of one child revealed an interstitial 4q13.3 microdeletion, 1.56 Mb in size. FISH analysis confirmed the deletion in the proband and identified the same deletion in her affected sib and mother, while it was not detected in a healthy sib. Deletion includes *ADAMTS3*, *ANKRD1*7, *COX18*, *GC*, and *NPFFR2* protein-coding genes.

**Conclusions:**

Our findings suggest that 4q13.3 microdeletion is a cause of a recognizable phenotype of three affected individuals. The detected microdeletion is the smallest interstitial deletion in 4q13. We highlight *ADAMTS3*, *ANKRD17* and *RNU4ATAC9P* as candidate genes for intellectual disability, growth retardation and congenital heart defect.

## Background

Structural chromosome alterations can lead to various clinical features, the most common being intellectual disability and congenital anomalies [1, 2]. Deletion of a short (p) arm of chromosome 4, causing Wolf-Hirschhorn syndrome characterized by intellectual disability, the Greek warrior helmet appearance of the nose and forehead, growth delay and seizures [3], is well described in the literature, while deletions involving the long (q) arm of chromosome 4 are poorly investigated. Not many patients with deletions of the long arm of chromosome 4 are described in the literature [4–9]. Moreover, even fewer patients have been reported with small alterations in one particular region of chromosome 4 instead of large deletions or duplications. The use of chromosomal arrays allows to detect submicroscopic alterations and subsequently through genotype- phenotype correlations and gene function analysis create the possibility to identify candidate genes, responsible for specific clinical features.

We present three patients with a 4q13.3 deletion in an attempt to characterize clinical manifestation of this rare chromosomal alteration.

## Case presentation

### Clinical findings

**Fig. 1:**
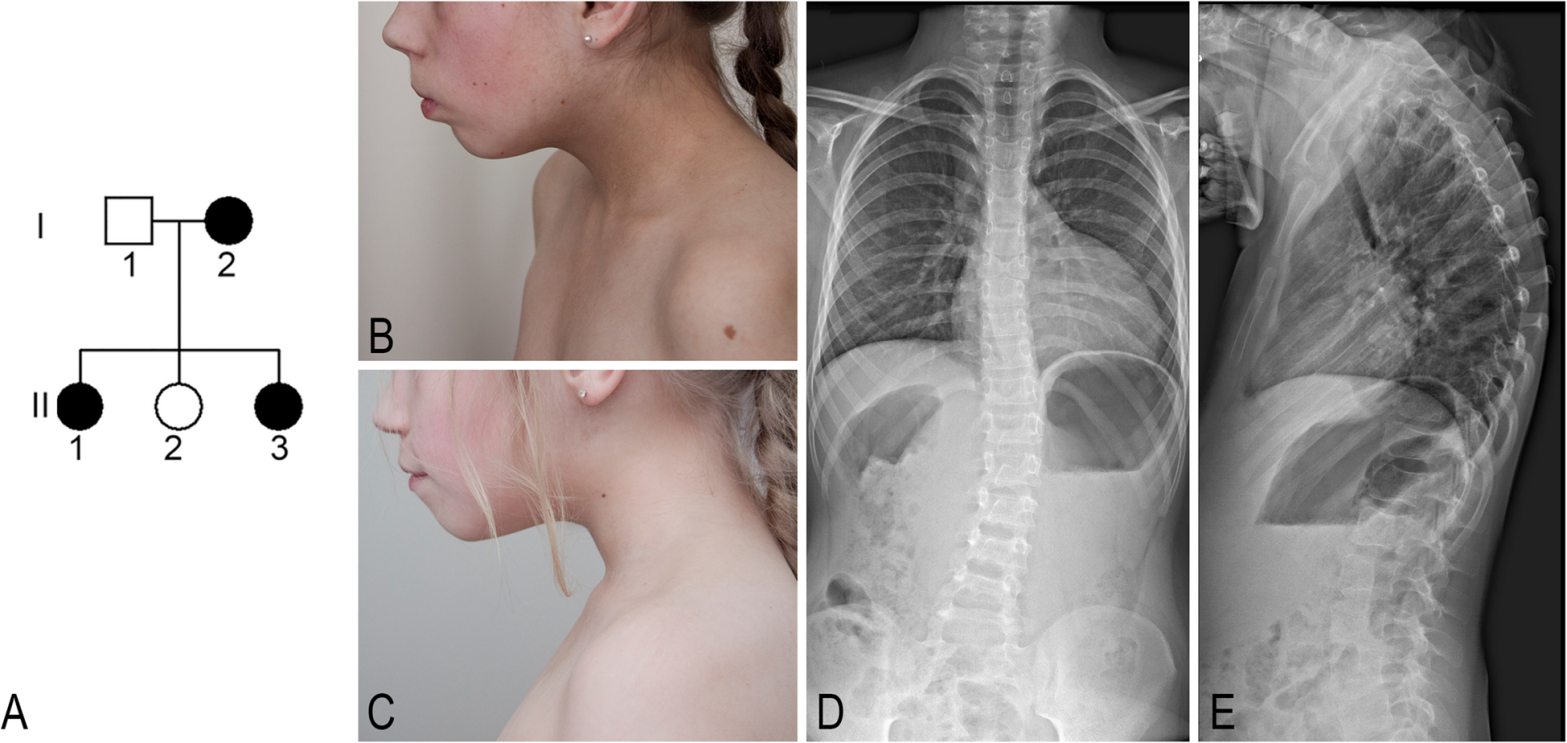
**a**. The genealogy of the family. The black symbol denotes affected individuals. **b**. Side facial view of patient 1 at age of 14 years. **c**. Side facial view of patient 2 at age of 9 years. **d**-**e**. Chest X-ray of patient 2 at age of 10 years showes “S” shaped II° scoliosis of the thoracolumbar spine

We report on three affected individuals in two generations of the Lithuanian family: two daughters, aged 19 years and 14 years, and their mother aged 43 years (Fig. 1a).

#### Patient 1 (DECIPHER 321792)

The patient is a 19-year-old female, the first child of non-consanguineous Lithuanian parents (Fig. 1a, individual II-1; Fig. 1b). She was born at the 41st-42nd gestational week by cesarean delivery due to signs of fetal asphyxia. Her birth weight was 2700 g (<3rd centile), her length was 48 cm (3rd –10th centile), and Apgar scores at 1, 5 and 10 min were 8, 9 and 10. Psychomotor developmental delay was noticed from infancy. According to the medical documentation, the girl’s height was below 3rd centile (130 cm and 138 cm at age of 15 and 16 years, respectively), weight was also below 3rd centile (28 kg and 36 kg at age of 15 and 16 years, respectively). At the age of 19 years, her head circumference was 51 cm (<3rd centile), her height was 147 cm (<3rd centile), her weight was 45 kg (<3rd centile), and her weight/height dependence was 75th–90th centile. She was found to have mild intellectual disability, microcephaly, micrognathia, upslanted palpebral fissures, coarse facial features, short neck, wide chest, spine deformation, and delayed puberty (according to Tanner B2, Ph2). An echocardiogram revealed an abnormality of the aortic valve and atrial septal defect with atrial septal aneurysm. No endocrine pathology was found, and bone age corresponded to chronological age. A chest X-ray showed S-shaped scoliosis of the thoracolumbar spine and no deformation of the ribs. No abnormalities were detected in a neck X-ray. An audiogram showed a mild neurosensory hearing impairment. Abdominal and renal ultrasound and brain MRI were normal. The patient’s IQ was measured at age 19 using the Wechsler Adult Intelligence Scale-III [10]: total IQ 56, verbal IQ 56, and non-verbal IQ 54.

#### Patient 2

The patient is a 14-year-old female and the third child in the family (Fig. 1, individual II-3; Fig. 1c–e). She was born at term naturally, following a normal pregnancy. Her birth weight was 2800 g (3rd centile), her length was 47 cm (3rd centile), and Apgar scores at 1 and 5 min were 9 and 10. After birth, an intrauterine infection, cystitis, urinary tract infection, CNS irritation syndrome, and bilateral subependymal cyst in the lateral ventricles of the brain were diagnosed. Psychomotor development delay was noticed from infancy; she started to walk at the age of 17 months. At the age of 8 years, she was diagnosed with hypopituitarism. According to the medical documentation, the girl’s height was below 3rd centile (123 cm and 135 cm at age of 10 and 12 years, respectively), and her weight was at 3–10 centiles (29 kg and 32 kg at age of 10 and 12 years, respectively). The patient’s psychological evaluation at age 13 yielded with full scale of 54 on Wechsler Intelligence Scale for Children (WISC-III-LT) [11], verbal IQ - 51, non-verbal IQ - 64. At the age of 14 years, her head circumference was 51.5 cm (3rd centile), her height was 141.5 cm (<3rd centile), her weight was 34 kg (<3rd centile), and her weight/height dependence was in the 25th–50th centile. She was found to have intellectual disability, irregular posture, short neck, upslanted palpebral fissures, coarse facial features, micrognathia, widely spaced nipples, and clinodactyly of the first and second digits of the foot. Deformation of the third digit of the foot was a consequence of trauma. She suffers from frequent respiratory tract infections and anemia. An echocardiogram revealed an abnormality of the aortic valve. A chest X-ray showed S-shaped second degree scoliosis of the thoracolumbar spine, thoracic kyphosis, and incomplete closure of the vertebral arches of Th8 and S1. A neck X-ray showed osteochondrosis. A brain MRI revealed cavum septi pellucidi. An inner organ ultrasound showed hypoplasia of the right kidney. Hypermetropic astigmatism and strabismus in both eyes was also diagnosed. An audiogram showed mild neurosensory hearing impairment.

#### Patient 3

The mother of patients II-1 and II-3 is a 43-year-old female who is a child of non-consanguineous parents (Fig. 1, individual I-2). During puberty she wore an orthopaedic corset due to a spine deformation and an orthopaedic pad for a shorter right leg. Regular menses started at age of 16 years. At the age of 40 years her head circumference was 52 cm (3rd centile), her height was 149 cm, her weight was 52 kg, and her BMI was 23. She was found to have intellectual disability, coarse facial features, hypotelorism, irregular posture, and wide chest. She suffers from frequent back pain, which spreads to both legs. She was diagnosed with arterial hypertension, second degree blood pressure increase, hypertensive cardiopathy (impaired diastolic function, moderate risk group), and a small haemodynamically insignificant atrial septal defect. An audiogram showed a neurosensory hearing impairment. An inner organ ultrasound revealed gall bladder stones. A spinal MRI performed at the age of 41 showed straightened lordosis, first degree spondylolystesis of L5, spondylolysis, first degree deforming osteochondrosis in the lower part of the thoracic spine and L1–5, second-third degree deforming osteochondrosis in the L5–S1 vertebrae, and Th12–L1 partial stenosis and L5–S1 absolute stenosis of the spinal canal. The patient’s IQ was measured at age 42 using the Wechsler Adult Intelligence Scale-III [10]: total IQ 62, verbal IQ 61, non-verbal IQ 69.

### Genetic analysis

Cytogenetic analysis from a culture of peripheral blood lymphocytes was performed using conventional GTG-banding techniques at resolution of the 550-band level according to standard procedures [12].

Genomic DNA was extracted from the patients’ peripheral blood samples using phenol-chloroform extraction method. A whole genome SNP (single nucleotide polymorphism) microarray analysis was performed for the patient 1 to detect copy number variations using the HumanCytoSNP-12v2.1 BeadChip, as described [13]. The data was analyzed using GRCh37/hg19 annotation.

FISH analyses were carried out on blood lymphocytes culture using commercial probes BACs – RP11-373 J21 (orange) from 4q13.3 region and RP11-118 N21 (green) from 4p15.2 region which served as control probe (Illumina, San Diego, CA, USA). All FISH procedures were followed according to the manufacturer’s protocol. Fluorescent signals on metaphases and interphase nuclei were analysed using a Nikon 80i fluorescent microscope with CytoVision version 3.6 (Applied Imaging, UK).

### Genetic findings

**Fig. 2:**
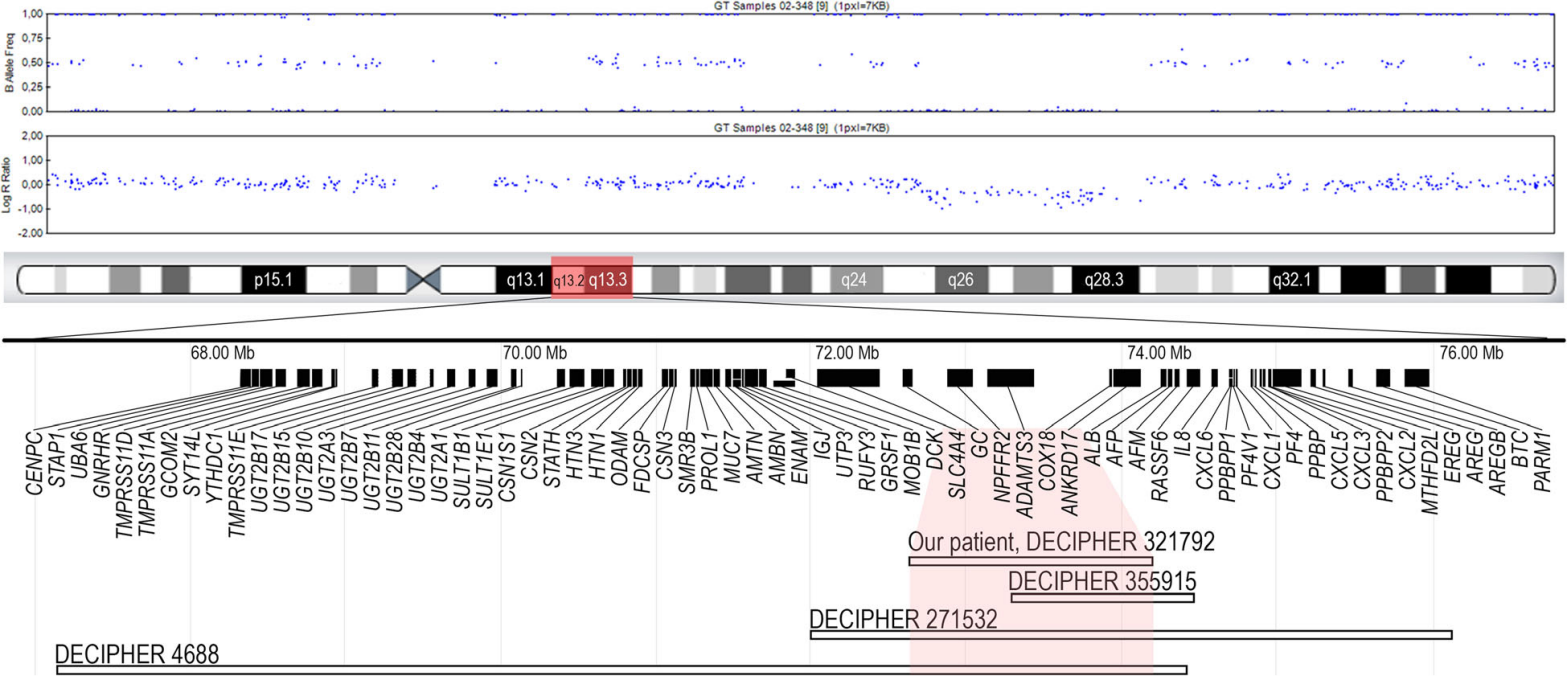
1.56 Mb deletion, arr[hg19] 4q13.3(72,647,749_74,208,199)×1 detected by SNP oligonucleotide microarray analysis and a schematic view of the genes. Horizontal white bars below represent deletions

Normal female karyotypes at 550 band resolution were observed in the affected sibs (patients 1 and 2). SNP oligonucleotide microarray analysis of the patient 1 revealed an interstitial 1.56 Mb deletion, arr[hg19] 4q13.3(72,647,749_74,208,199)×1 (Fig. 2). FISH analysis with RP11-373 J21 probe which overlaps part of *ADAMTS3* gene confirmed the deletion in the Patient 1 and identified the deletion at 4q13.3 in her affected sib (patient 2) and mother (patient 3). The deletion was not detected in healthy sib (II-2).

## Discussion and conclusions

We have provided a clinical and molecular characterization of a previously unreported 4q13.3 microdeletion, 1.5 Mb in size, detected in three affected individuals of a Lithuanian family. All patients presented with short stature, congenital heart defect, skeletal anomalies, minor facial anomalies, delayed puberty, and intellectual disability.

Most of the previously reported deletions involving the 4q13.3 region are large chromosomal alterations with a common loss of band 4q21, resulting in marked growth restriction, severe intellectual disability, and absent or severely delayed speech [5–9, 14]. The deletion detected in our patients is unique. There are no low copy repeats or other specific DNA elements flanking deleted region, therefore deletion can be caused either by nonhomologous end joining or microhomology-mediated replication-dependent recombination. Only four unrelated patients with overlapping 4q13.3 monosomy smaller than 10 Mb and not involving the 4q21 region have been reported in literature [8] or recorded in the DECIPHER database (271,532, 4688, and 355,915) [14] (Table 1). Quintela I et al. [8] have reported a de novo 6.85 Mb deletion at 4q13.2-q13.3 in the patient with psychomotor developmental delay, mild intellectual disability, behavioral disorder, short stature and facial anomalies, including a triangular craniofacial profile with a broad forehead, narrow and slightly upslanted palpebral fissures, a broad nasal tip and a prominent narrow chin with a dimple in its medial part [8]. Neurodevelopmental abnormalities including intellectual disability, delayed speech and language development, or autism were common for all patients with an interstitial deletion in 4q13.3. Short stature was found in the DECIPHER patient 4688 and the patient reported by Quintela I et al. [8, 14]. Additionally, the phenotype of DECIPHER patient 4688 is remarkable for strabismus and skeletal abnormalities which also manifested in our patients. The size of the common region in our patients and DECIPHER patients 271532, 4688, and 355915 is 905 kb. It includes three genes (*ADAMTS3, ANKRD17, COX18*) and four pseudogenes (*HNRNPA1P67*, *RNU4ATAC9P*, *RNU6ATAC5P*, *HMGA1P2*).

**Table 1:** Clinical features of the patients with overlapping 4q13.3 microdeletion

	Decipher 271532	Decipher 4688	Decipher 355915	Quintela I et al., 2015 Patient 2 [8]	This report		
**Proximal breakpoint (hg19)**	72,006,446	67,129,189	73,303,180	68,207,272	72,647,749		
**Distal breakpoint (hg19)**	76,140,123	74,425,009	74,459,331	75,021,494	74,208,199		
**Cytoband**	4q13.1q13.3	4q13.1q13.3	4q13.3	4q13.1q13.3	4q13.3		
**Size of deletion, Mb**	4.13	7.30	1.16	6.81	1.56		
**Inheritance**	De novo	De novo	NA	De novo	Maternal	Maternal	NA
**Gender**	F	M	F	F	F	F	F
**Age**	NA	NA	NA	11 yr.	19 yr.	14 yr.	43 yr.
**Neurologic**	ID	ID, delayed speech and language development	ID, autism	Mild ID	Mild ID	Mild ID, cavum septi pellucidi	Mild ID
**Head and face**	NA	NA	NA	Triangular face, broad forehead, pointed chin with chin dimple	Microcephaly, coarse face	Coarse face	Coarse face
**Eyes**	NA	Strabismus	NA	Slightly upslanted palpebral fissures	Upslanted palpebral fissures	Upslanted palpebral fissures, hypermetropic astigmatism, strabismus	Hypotelorism
**Ears**	NA	NA	NA	N	Mild neurosensory hearing impairment	Mild neurosensory hearing impairment	Neurosensory hearing impairment
**Nose, mouth**	NA	NA	NA	Broad nasal tip	Micrognathia	Micrognathia	NA
**Skeletal**	NA	Short stature, abnormality of the lower limb, clinodactyly of the 5th finger	NA	Short stature	Short stature, short neck, thoracolumbar scoliosis	Short stature short neck, thoracolumbar scoliosis, malformed vertebrae, clinodactyly of toes	Short stature, spine deformation, osteochondrosis, stenosis of the spinal canal
**Cardio-vascular**	NA	NA	NA	N	Abnormality of the aortic valve, atrial septal defect with atrial septal aneurysm	Abnormality of the aortic valve	Atrial septal defect, primary arterial hypertension, hypertensive heart disease
**Endocrine**	NA	NA	NA	NA	Delayed puberty	Hypopituitarism, delayed puberty	NA
**Genito-urinary**	NA	NA	NA	NA	NA	Hypoplasia of the right kidney	NA

*Abbreviations*: *F* female, *M* male, *yr*. years, *ID* Intellectual disability, *N* Normal, *NA* Not Available

The *ADAMTS3* gene encodes an enzyme belonging to the metalloproteases family, which is responsible for procollagen I and II processing in various tissues [15]. It is highly expressed in cartilage formation during embryogenesis and after birth. Studies have shown that *Adamts3* was also expressed in developing mouse connective tissues, especially tendon and bone [16]. *ADAMTS2* mutations in humans lead to characteristic craniofacial changes and decreased growth [16]. Therefore it could be associated with such clinical features presented in our patients as skeletal anomalies and short stature. The same study discovered *Adamts3* expression in several regions of developing mouse brains, including the cerebral cortex [16].

*ANKRD17* encodes an ubiquitously expressed protein that was found to be essential to vascular integrity during embryogenesis. *Ankrd17*-deficient mice developed various heart defects and haemorrhages and a decrease in vascular smooth muscle cells leading to death [17]. Deletion of this gene could be related to the various heart defects described in our patients. ANKRD17 is also thought to play an essential role in DNA replication [18]. Study suggests that it is involved in DNA pre-replication complex formation as well as remodelling and transcribing of chromatin [18]. A loss of or decrease in ANKRD17 blocks DNA replication and inhibits cell cycle progression [18]. However, more studies are required to determine ANKRD17 protein function more precisely.

The role of the *COX18* gene and pseudogenes within the deletion is still poorly understood. Pseudogenes may be transcribed into RNA and can be processed into short interfering RNAs that regulate coding genes. It is known that the *RNU4ATAC9P* pseudogene and its parental gene *RNU4ATAC* could form a regulatory pair that can influence each other [19]. Mutations in the *RNU4ATAC* cause the autosomal recessive Roifman Syndrome (MIM#616651), which is characterized by growth retardation, cognitive delay, spondyloepiphyseal dysplasia, and antibody deficiency [20]. Still, the association of genes within the deletion to human disorders remains to be elucidated.

Our report presents detailed molecular and phenotypic characteristics of three affected individuals providing new data supporting future genotype-phenotype studies and identification of candidate genes possibly responsible for specific clinical features. The detected microdeletion in affected individuals of the presented family is the smallest interstitial deletion in 4q13. We highlight *ADAMTS3*, *ANKRD17* and *RNU4ATAC9P* as candidate genes for intellectual disability, growth retardation and a congenital heart defect. Further contributions of genotype-phenotype descriptions are required for the delineation of the complete spectrum of findings that may be seen associated with microdeletions in 4q13.3 region and clarifying the role of each of the deleted genes.

## Data Availability

The datasets generated and analyzed during the current study are available in the Gene Expression Omnibus repository, https://www.ncbi.nlm.nih.gov/geo/query/acc.cgi?acc=GSE147730*.*

## Abbreviations

*ADAMTS3*: ADAM Metallopeptidase With Thrombospondin Type 1 Motif 3
*ADAMTS2*: ADAM Metallopeptidase With Thrombospondin Type 1 Motif 2
*ANKRD1*7: Ankyrin Repeat Domain 17
*COX18*: Cytochrome C Oxidase Assembly Factor COX18
*GC*: Group-specific component
*HNRNPA1P67*: Heterogeneous Nuclear Ribonucleoprotein A1 Pseudogene 67
*MGA1P2*: High Mobility Group AT-Hook 1 Pseudogene 2
*NPFFR2*: Neuropeptide FF Receptor 2
p: Short arm of chromosome
q: Long arm of chromosome
*RNU4ATAC*: RNA, U4atac Small Nuclear (U12-Dependent Splicing)
*RNU6ATAC5P*: RNA, U6atac Small Nuclear 5, Pseudogene
*RNU4ATAC9P*: RNA, U4atac Small Nuclear 9, Pseudogene
SNP: Single nucleotide polymorphism
